# Alcohol Consumption and Blood Pressure Among Older Couples: Negative Implications of Drinking Concordance

**DOI:** 10.1093/geroni/igaa057.2235

**Published:** 2020-12-16

**Authors:** Kira Birditt, Angela Turkelson, Courtney Polenick, James Cranford, Frederic Blow

**Affiliations:** 1 University of Michigan, Ann Arbor, Michigan, United States; 2 University of Michigan Medical School, Ann Arbor, Michigan, United States

## Abstract

Couples who are similar in their drinking behaviors (i.e., concordance) often report better quality and longer marriages. These patterns of drinking may have negative implications for blood pressure, however, as individuals age. The present study examined the effects of alcohol use on blood pressure among couples and whether the associations varied by age. Participants included 2311 married/cohabiting couples (4487 individuals; aged 52 to 92) followed from 2006 to 2014 in the Health and Retirement Study who reported their average drinks per week and had their blood pressure measured. Multilevel models revealed older men (aged 60+) who drank more had higher blood pressure irrespective of wives drinking whereas for younger men the link between their own drinking and increased blood pressure was greater when wives drank more. Although concordance may be beneficial for marriage, there may be a downside to drinking concordance for health especially among middle aged men. Part of a symposium sponsored by Dyadic Research on Health and Illness Across the Adult Lifespan Interest Group.

